# Microgravity reshapes bacteriophage–host coevolution aboard the International Space Station

**DOI:** 10.1371/journal.pbio.3003568

**Published:** 2026-01-13

**Authors:** Phil Huss, Chutikarn Chitboonthavisuk, Anthony Meger, Kyle Nishikawa, R. P. Oates, Heath Mills, Olivia Holzhaus, Srivatsan Raman

**Affiliations:** 1 Department of Biochemistry, University of Wisconsin-Madison, Madison, Wisconsin, United States of America; 2 Department of Bacteriology, University of Wisconsin-Madison, Madison, Wisconsin, United States of America; 3 Microbiology Doctoral Training Program, University of Wisconsin-Madison, Madison, Wisconsin, United States of America; 4 Rhodium Scientific Inc., Houston, Texas, United States of America; 5 Department of Chemical and Biological Engineering, University of Wisconsin-Madison, Madison, Wisconsin, United States of America; Monash University, AUSTRALIA

## Abstract

Bacteriophage–host interactions play a fundamental role in shaping microbial ecosystems. While extensively studied on Earth, their behavior in microgravity remains largely unexplored. Here, we report the dynamics between T7 bacteriophage and *Escherichia coli* in microgravity aboard the International Space Station (ISS). Phage activity was initially delayed in microgravity but ultimately successful. We identified de novo mutations in both phage and bacteria that enhanced fitness in microgravity. Deep mutational scanning of the phage receptor binding domain revealed striking differences in the number, position, and mutational preferences between terrestrial and microgravity conditions, reflecting underlying differences in bacterial adaptation. Combinatorial libraries informed by microgravity selections yielded T7 variants capable of productively infecting uropathogenic *E. coli* resistant to wild-type T7 under terrestrial conditions. These findings help lay the foundation for future research on the impact of microgravity on phage–host interactions and microbial communities and the terrestrial benefits of this research.

## Abstract 2

Phage-host interactions have a role shaping microbial ecosystems, but their behavior in microgravity remains largely unexplored. In this study, experiments aboard the ISS reveal distinct evolutionary adaptations in T7 bacteriophage and E. coli, revealing phage variants effective against resistant terrestrial pathogens.

## Introduction

The interaction between bacteriophages (or “phages”) and their bacterial hosts plays a fundamental role in shaping microbial ecosystems both in humans and in the environment [1–6]. Phages act as major drivers of bacterial diversity and evolutionary change in their bacterial prey. These interactions are determined not only by the molecular compatibility of phages and hosts but also by the larger physical context in which infections take place, with factors such as fluid mixing, nutrient gradients, and the underlying physiology of both the bacterial cell and the phage exerting a strong influence [7–9]. Although phage–host interactions have been extensively studied in terrestrial ecosystems, the impact of microgravity on these interactions has yet to be fully investigated. Studying phage–host interplay in microgravity may reveal new mechanisms with relevance both in space and on Earth.

Microgravity is the near-weightless condition in orbit and alters both the physical transport processes and the physiological states that shape phage predation and bacterial growth, creating an environmental niche distinct from any found terrestrially. At the physical level, phage particles typically diffuse randomly through liquid until they collide with a susceptible bacterial cell, at which point short-range forces such as van der Waals interactions and electrostatic attraction enable irreversible adsorption and subsequent injection of the phage genome [7,10]. Under normal gravity on Earth, this process is enhanced by natural convection. Density- and temperature-dependent buoyancy drives fluid circulation and sedimentation continually redistributes phages, nutrients, and metabolic byproducts, thereby increasing the probability of phage–host encounters. In microgravity, however, materials of differing densities fail to separate, convection currents driven by gravity no longer form, and nutrient molecules as well as motile bacteria experience restricted diffusion and disrupted motility [11–16].

The absence of gravity also profoundly reshapes bacterial physiology, imposing stresses that reverberate through gene regulation and metabolism [16–19]. Numerous studies have shown that microgravity conditions increase biofilm formation and elevate metabolic rates [20–22], while the reduced mixing of the surrounding medium limits the removal of metabolic waste and the replenishment of essential nutrients, thereby inducing the overexpression of starvation-associated genes, altering membrane transport processes, and driving global adjustments in cellular homeostasis [23,24]. Bacteria confronted with these conditions may adapt by altering their proteome, including modifying outer-membrane components that act as phage receptors, which can directly influence susceptibility to infection and the efficiency of phage adsorption [15,19,25]. Taken together, these physical and physiological perturbations underscore that microgravity constitutes a distinct and multifaceted environment capable of significantly modifying phage–host dynamics, with consequences that are likely to inform our understanding of microbial community behavior not only in extraterrestrial habitats but also in engineered and extreme terrestrial ecosystems.

In this study, we investigated how microgravity affects interactions between T7 bacteriophage and non-motile *Escherichia coli* BL21 aboard the International Space Station (ISS). Our evaluation of short-term (hours) and long-term (23 days) incubation of phage and host in microgravity showed significant differences in phage and bacterial viabilities and phage activity compared to terrestrial controls. Phages accumulated many de novo mutations over time that may enhance receptor binding or phage infectivity, while bacteria acquired de novo mutations in genes that may enhance fitness in microgravity and counter phage predation. Deep mutational scanning (DMS) of the phage receptor binding protein (RBP) in microgravity revealed a fitness landscape significantly different from our terrestrial experiments, suggesting substantial differences in the host receptor profile and selection pressure under microgravity. Notably, a combinatorial library of RBP variants enriched in microgravity exhibited a significant improvement in activity against terrestrial uropathogenic *E. coli*, while a similar library derived under terrestrial conditions showed no improvement, highlighting microgravity as a source of insights into phage–host dynamics with relevance to Earth. Overall, our findings help lay a foundation for future research into the impact of phage–host interactions on microbial communities in microgravity and in the context of space exploration.

## Results

### Design of experiments for the International Space Station

**Fig 1:**
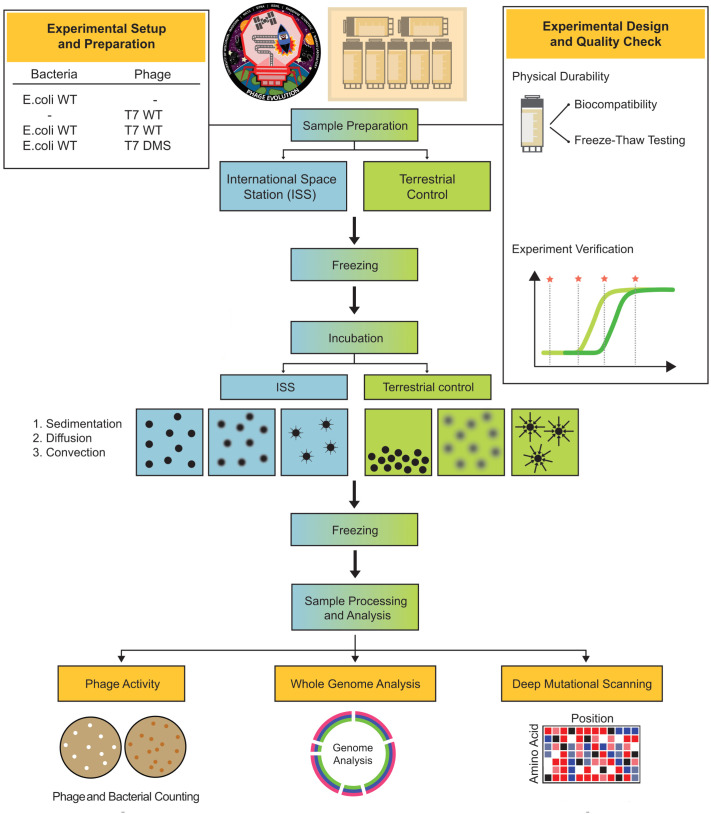
Experimental design to evaluate microgravity interactions on the ISS. Samples were prepared on Earth, with quality checks to ensure cryovial integrity and prevent leakage during freeze–thaw cycles. Identical sets were frozen, then thawed and incubated either in microgravity on the ISS (left) or terrestrially (right) for defined intervals. All samples were re-frozen and later analyzed on Earth for phage and bacterial titers, whole-genome sequencing, and deep mutational scanning of the T7 receptor binding protein tip domain.

We prepared two identical sets of 32 sealed cryovial tubes containing experimental samples: one designated for incubation in microgravity, and the other designated for terrestrial incubation (Fig 1). Each set was divided into four prepackaged groups of eight tubes for incubation at 37 °C. Three groups were incubated for short-term time points (1, 2, and 4 hours), and one group for a long-term time point (23 days).

Each short-term group included three replicates of T7 and *E. coli* BL21 mixed at a phage to host ratio (multiplicities of infection or MOIs) of 10^−6^ and 10^−4^ and two samples with either T7 phage only or *E. coli* only. All bacterial samples contained 4 mL of log-phase (OD_600_ ~0.4) *E. coli* with an estimated titer of ~1–2 × 10^8^ CFU/mL. The initial bacterial concentration and MOI were selected to allow for measurable changes in phage titer after incubation between timepoints, accounting for anticipated cell and phage viability loss due to freeze–thawing as part of delivering samples to the ISS and returning them terrestrially. No cryoprotectant was added to any cultures. To isolate the effects of microgravity, we used a non-motile *E. coli* strain, removing the variable of host motility enhancing fluid mixing. The 23-day group included three replicates of T7 phage and *E. coli* mixed at an MOI of 10^−4^, three replicates of T7 DMS library mixed with *E. coli* at an MOI of 10^−2^, and two samples of either T7 only and *E. coli* only. The higher MOI for the DMS group compensated for the lower abundance of individual variants. The DMS library comprises 1,660 T7 variants, each with a single amino acid substitution in the tip domain of the RBP that has been previously tested under terrestrial conditions [26]. RBPs are central to phage biology, as they dictate host recognition, adsorption, and ultimately host range [27–32]. Their ability to interact with diverse bacterial surface molecules and to evolve rapidly through genetic variation makes them key determinants of phage adaptability and therapeutic potential. Given this central role, RBPs are a particularly compelling target for DMS to decode the sequence–function rules underlying host specificity and infection efficiency.

The cryovial containers passed biocompatibility, leak testing, and experimental validation (see S1 Data) to ensure sample integrity and comply with NASA safety standards. All samples were prepared on Earth by mixing phage and bacteria in Rhodium cryovials and immediately freezing them at −80 °C. Frozen samples were shipped to NASA’s Wallops Flight Facility 24 days before launch and transported to the ISS aboard the Northrop Grumman NG-13 Cygnus rocket. Samples were incubated at 37 °C in microgravity for the duration of the relevant time point, then refrozen at −80 °C, transported back to Earth, and delivered to our laboratory. We then thawed the samples, measured phage and bacterial titers, sequenced their genomes, and analyzed the DMS library (Fig 1). We recorded the duration of freezing and incubation aboard the ISS and evaluated the second set of samples terrestrially using the same incubation and freezing times. Terrestrial Incubation was performed without shaking. An asynchronous ground control is standard practice for space biology flight experiments because microgravity and terrestrial samples cannot be incubated in parallel accurately as actual time points on the ISS are adjusted to accommodate astronaut scheduling and real time tracking of samples is not possible.

### Bacteriophage T7 activity is reduced in microgravity

Under normal terrestrial conditions with shaking at 37 °C, the T7 phage infects and lyses *E. coli* BL21 within 20–30 min and produces 100–200 progeny phages [33,34]. We hypothesized that in microgravity, reduced fluid mixing would slow the infection cycle by limiting productive encounters between phages and bacteria. Additionally, microgravity-induced stress might disrupt host homeostasis, alter receptor expression, or interfere with intracellular processes, impeding successful phage replication. To test this hypothesis, we measured phage and bacterial titers after 1-, 2-, and 4-hours, as well as after 23 days of incubation. Because the extent of phage replication delay in microgravity was unknown, this approach allowed us to capture a broad range of possible delays.

**Fig 2:**
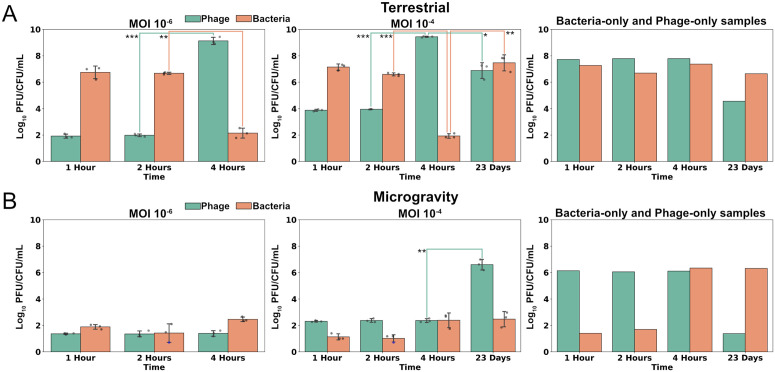
Bacteriophage T7 growth is inhibited by microgravity. The titer of phage (green) and bacteria (orange) samples (log_10_ Plaque Forming Units or Colony Forming Units, PFU/CFU/mL) after **(A)** terrestrial incubation or **(B)** incubation in microgravity mixed at a pre-freeze MOI of 10^−2^ (left), 10^−4^ (middle), or incubated separately as phage-only or bacteria-only samples (right). Bars show mean ± SD; triplicate samples shown as points, with blue indicating values at the limit of detection. The significance between adjacent time points was assessed by two-sample *t* test (*p < 0.05, **p < 0.01, ***p < 0.001). The data underlying this Figure can be found in S1 Data.

Phage and bacteria were co-cultured at pre-freeze MOIs of 10^−6^ and 10^−4^ for short-term incubation time points (1-, 2-, and 4-hours) and 10^−4^ for long-term incubation (23-days). A significant increase in phage titer and decrease in bacterial host titer between incubation time points would indicate successful phage activity. We did not analyze differences in titer from the initial MOI under the assumption sample freezing would impact viability and instead interpret results between timepoints to determine if phage can successfully replicate. Under terrestrial conditions phage titers increased significantly by 5–7 logs and bacterial titers decreased significantly by 4–5 logs after four hours, regardless of the MOI (Fig 2A). Phage infection thus occurred between two and four hours for the terrestrial samples, indicating the experimental conditions delayed the infection cycle by approximately two hours while allowing for successful phage replication.

Under microgravity conditions, we observed no increase in phage titer at any short-term incubation time points at either MOI, but a significant 4-log increase at the 23-day time point (Fig 2B). This result indicates microgravity did not prevent productive infection and lysis but delayed it to some point past the four-hour time point. The persistence of bacteria at the 23-day time points (approximately 10^7^ CFU/mL for the terrestrial samples and 10^2^ CFU/mL for microgravity samples) also suggests that a phage-resistant bacterial population emerged in both conditions.

Bacterial titer without phage remained stable at early time points under terrestrial incubation but fell 6–7 logs at the 1- and 2-hour time points in microgravity. One possibility, though unproven, is that the absence of cryoprotectant contributed to reduced bacterial viability during freeze–thaw. We cannot exclude an additional role for microgravity-related stress, but emphasize that further experiments will be needed to disentangle these effects. Phage without bacteria saw a 2-log decrease in viable titer in early time points in microgravity compared to terrestrial incubation, but otherwise appeared more stable than bacteria, except for the 23-day time point, where we observed a 4- and 7-log fold decrease in phage titers terrestrially and in microgravity, respectively. Phages are known to lose stability and decay over time without a propagating host [35–37]. Although freeze–thaws complicate accurately determining a decay rate, this effect appeared more pronounced in microgravity.

These experiments demonstrate that microgravity challenges both phage and bacterial viability. While phage infectivity is delayed compared to terrestrial conditions, phages ultimately overcome this barrier and successfully infect their hosts. Future studies targeting intermediate time points will be critical for defining the precise latent period under microgravity.

### Enriched mutations are distributed broadly in T7 phage

**Fig 3:**
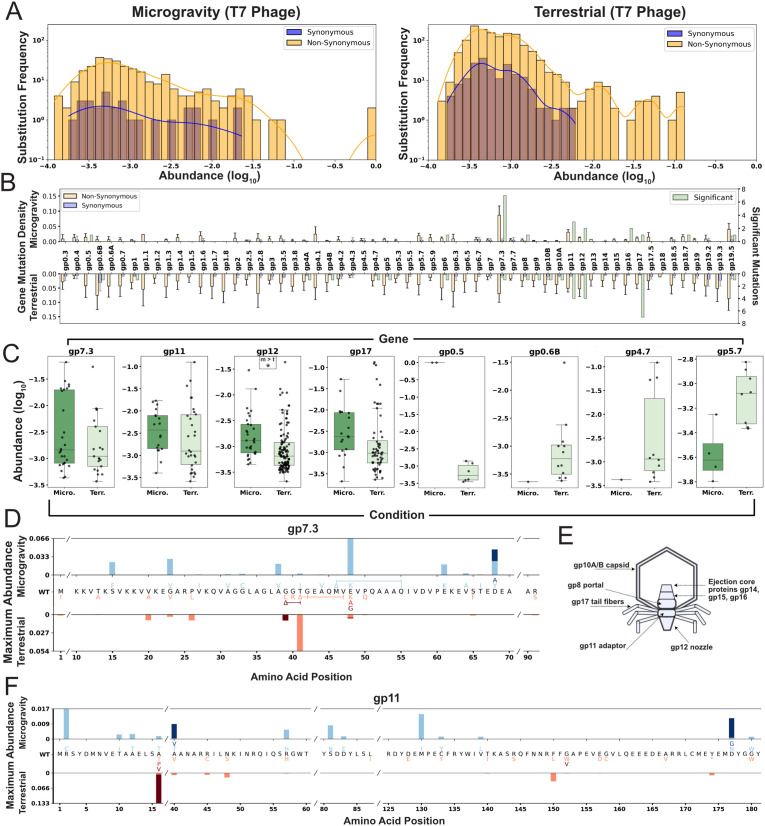
Enriched mutations are distributed broadly in T7 phage. **(A)** Substitution frequency and abundance of phage de novo synonymous (blue) and non-synonymous substitutions or frameshifts (yellow) after microgravity (left) or terrestrial (right) incubation. **(B)** Average gene mutation density ±SD for de novo synonymous (blue) and non-synonymous (yellow) substitutions after microgravity (top) and terrestrial (bottom) incubation. Green bars show the number of significant mutations per gene from **(A)**. **(C)** Log_10_ abundance of mutations in selected phage genes after microgravity (left, dark green, Micro.) and terrestrial (right, light green, Terr.) incubation. Significance shown as * (adjusted *p* < 0.05, one-tailed *t* test with FDR). **(D)** Maximum substitution abundance in *gp7.3* after microgravity (top, blue shading) or terrestrial (bottom, red shading) incubation. Wild-type (WT) sequence shown center with substitutions shaded matching bar color. Deletions shown as Δ; slashes (/) in WT sequence mark omitted unmutated regions. **(E)** Diagram of selected structural genes. *Gp7.3* is excluded due to an uncertain structural role. **(F)** Maximum substitution abundance in *gp11*, displayed as in **(D)**. The data underlying this Figure can be found in S1 Data.

Next, we sought to identify mutations in the phage or bacterial genome that influenced phage-host interactions under microgravity. We performed whole-genome sequencing (WGS) of T7 and *E. coli* BL21 before and after incubation, using pre-incubation genomes as references to identify de novo mutations in the 23-day samples from each condition to ensure both phage and bacterial populations had ample time to propagate. To determine whether de novo non-synonymous substitutions or frameshifts in T7 were significantly enriched, we compared the pooled frequencies of abundant non-synonymous mutations to the distribution of synonymous de novo substitutions in each condition (Mann–Whitney *U* test, FDR-adjusted *p* < 0.05; Fig 3A). To assess whether specific genes had significantly more non-synonymous substitutions than other genes, we calculated this mutation density for each gene and compared it to the average mutation density per condition (one-tailed *t* test, FDR-adjusted *p* < 0.05, one-sided 95% CI; Figs 3B and S1). Finally, we compared the gene-level distribution of non-synonymous mutations between microgravity and terrestrial conditions (Mann–Whitney *U* test, FDR-adjusted *p* < 0.05) to identify genes with condition-specific enrichment of these mutations (Figs 3C and S2A).

Significantly enriched (*p* < 0.05) phage substitutions were found across both structural and non-structural proteins under terrestrial and microgravity conditions (Fig 3B). In microgravity, gene product (*gp*) 7.3 and *gp11* exhibited significantly more de novo non-synonymous substitutions than other genes (Figs 3B and S1A). Mutation density was overall higher terrestrially and no gene showed significant enrichment compared to others terrestrially (S1B Fig). Although *gp7.3* is not fully characterized, it is considered essential for T7 infectivity in *E. coli* BL21 under terrestrial conditions [38]. This small 99-amino-acid protein may function as a scaffolding protein or contribute to host adsorption, though its role in the mature virion remains uncertain [39–41]. *gp7.3* harbored seven significantly enriched substitutions in microgravity, the highest number observed in any gene under that condition. These substitutions were distributed throughout the protein (Figs 3D and S3), with four notable changes (E48K, E61K, D68Y, D68A) involving substantial shifts away from negatively charged residues. The only significantly enriched mutation in *gp7.3* terrestrially was a six-amino-acid deletion spanning G42 to V47. The region from G39 to Q50 contained a dense cluster of substitutions and in-frame deletions, including a 3-amino-acid deletion (G39-T41) terrestrially and a deletion from M46 to Q55 in microgravity, all occurring in a region of the protein predicted to be unstructured (S3 Fig). The high number of enriched substitutions and recurring in-frame deletions in this small protein suggest that *gp7.3* is both structurally flexible and critical for phage activity in both environments.

*gp11* is an adaptor protein within the T7 tail that connects the portal protein *gp8*, the nozzle protein *gp12*, and the six subunits of the tail fiber protein *gp17* (Fig 3E) [38,40,42]. Enriched substitutions were distributed throughout *gp11*, spanning both exposed and buried residues (Figs 3F, S4A, and S4B). One significantly enriched substitution, R2C, arose independently twice in microgravity and is located in a flexible region capable of directly interacting with *gp17* tail fibers (S4C and S4D Fig). These findings suggest that the substitutions may influence phage fitness by altering *gp11*’s structure or stability rather than through direct interaction with the bacterial host.

Comparison of mutation abundance revealed that de novo non-synonymous substitutions were significantly more prevalent in the nozzle protein *gp12* after incubation in microgravity than under terrestrial conditions, suggesting a more prominent role for this protein in microgravity (Fig 3C). Of the six individually enriched non-synonymous substitutions identified across both conditions, five involved changes toward positively charged residues (Q184R, R205H, Q242R, K404R, and W707R). These substitutions were distributed throughout the protein, with three more likely contributing to host interactions (S5 Fig). Specifically, R205 is surface-exposed and positioned near the host, Q242 lies close to the terminus of the DNA delivery channel, and Q184 faces directly toward the host. The charge shifts and spatial distribution of these substitutions highlight the functional importance of *gp12* in enhancing phage fitness under both terrestrial and microgravity conditions.

Several other significantly enriched substitutions were particularly notable. In microgravity, the V26I substitution in *gp0.5* was the only mutation to sweep the entire phage population—and did so independently in two replicates—indicating a strong fitness advantage. *gp0.5* is an uncharacterized class I gene, potentially associated with the host membrane due to the presence of a putative transmembrane helix [22]. Under terrestrial conditions, the T115A substitution in *gp4.7* was significantly enriched and highly abundant across all three replicates. No mutations were detected in this gene under microgravity, suggesting selection pressure may be unique to terrestrial conditions. Although the function of gp4.7 remains unknown, BLASTP analysis identified homologs with ~40% similarity to putative HNH endonucleases in *Klebsiella* and *Pectobacterium* phages [43].

Lastly, numerous significantly enriched substitutions were found in the tail fiber *gp17*, particularly under terrestrial conditions (Fig 3B). In both environments, substitutions were concentrated in the C-terminal tip domain, with repeated mutations at D540 and neighboring residues. This region is a known determinant of host range and infectivity in terrestrial *E. coli* strains [26], and these results suggests continued importance during prolonged incubation in both gravity conditions.

### Enriched bacterial mutations reflect phage-mediated selection

**Fig 4:**
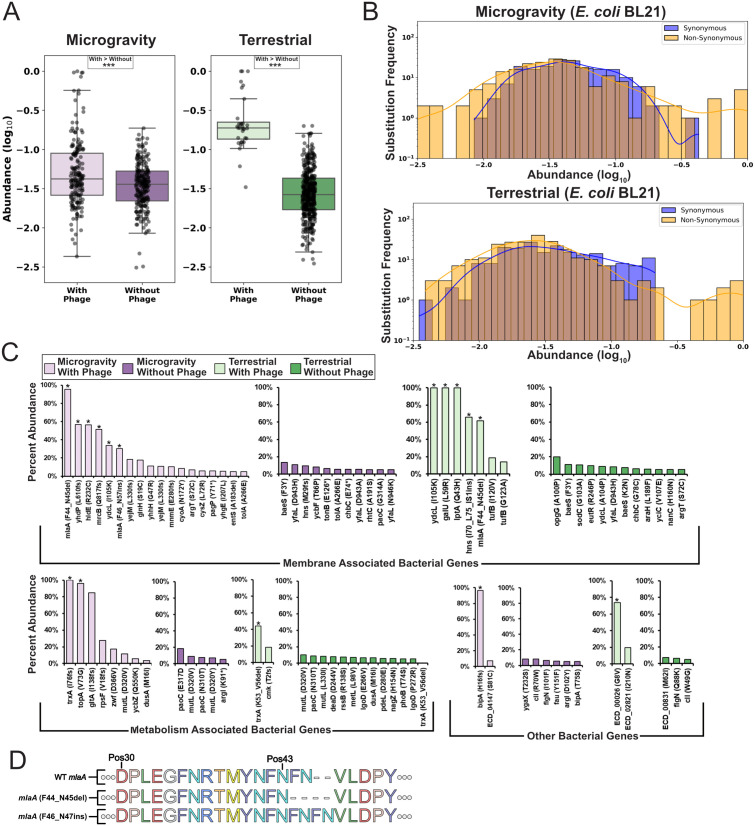
Enriched bacterial mutations reflect phage-mediated selection. **(A)** Boxplots of log_10_ abundance for de novo non-synonymous substitutions and frameshifts after microgravity (left) or terrestrial (right) incubation, comparing incubation with (light shading) and without (dark shading) phage. Significance assessed by Mann–Whitney *U* test (***p < 0.001), with “>” indicating the more abundant population. **(B)** Frequency for bacterial de novo synonymous (blue) and non-synonymous substitutions or frameshifts (yellow) after microgravity (top) or terrestrial (bottom) incubation. **(C)** Maximum abundance of *E. coli* BL21 non-synonymous substitutions or frameshifts (>5%) after incubation in microgravity with phage (light purple) or without phage (dark purple), and after terrestrial incubation with phage (light green) or without phage (dark green). Grouped by membrane-associated genes (top), metabolism-associated genes (bottom-left), or other genes (bottom-right). Mutations significantly enriched in (B) marked with stars. **(D)** Illustration of *mlaA* mutation effects. Deletions and insertions result in loss or repetition of phenylalanine and asparagine residues. The data underlying this Figure can be found in S1 Data.

De novo mutations in *E. coli* BL21 were significantly more abundant in samples mixed with phages than in those without phages under both terrestrial and microgravity conditions, indicating strong phage-driven selective pressure in both environments (Figs 4A and S6A–S6C, Mann–Whitney *U* test *p* < 0.001, Kaplan–Meier survival and log-rank statistical test *p* < 0.001). Sequencing of the 4-hour sample revealing no significant deviation in this population compared to the pre-incubation sample (Pearson’s *R* = 0.986, Jenson-Shannon divergence 0.0543, S6D and S6E Fig) and the 4-hour bacterial population was equally susceptible to T7 phage compared to the pre-incubation population (S6F Fig), indicating there was no bottlenecking effect during initial incubation that could cause lowly abundant mutations in the original population to appear de novo. Significantly enriched non-synonymous de novo bacterial substitutions and frameshifts were present in both conditions (Fig 4B, Mann–Whitney *U* test, FDR-adjusted *p* < 0.05, > 25% abundance), and pooling genes on Gene Ontology (GO) categories [44,45] revealed that most enriched genes were associated with membrane function or the regulation of metabolic process (Fig 4C).

Bacterial mutations significantly enriched (*p* < 0.05) only under microgravity were frequently associated with the outer membrane and cellular stress response. Notable examples include *hldE* (56.5%, R232C) associated with the synthesis of the LPS core [46]; *mrcB* (51.6%, Q817 frameshift), which plays a role in cell wall synthesis and permeability [47,48]; and *bipA* (96.5%, H16 frameshift, also known as *typA*) linked to LPS biosynthesis and temperature sensitivity, and previously associated with truncated LPS phenotypes [35–37]. The significant abundance of this mutation suggests that *bipA* might play a role in phage sensitivity in microgravity. *topA*, a DNA topoisomerase associated with stress response, was also significantly enriched in microgravity (96.1%, V73G) [49,50]. Additional mutations unique to microgravity included *gltA* (85%, I138 frameshift), a citrate synthase [51], and *rpsF* (27.7%, V18 frameshift), which encodes a 30S ribosomal protein [52]. Under terrestrial conditions, bacterial mutations significantly enriched in the presence of phage included *galU* (100%, L59R), involved in UDP glucose metabolism and associated with O-polysaccharide in other strains [53,54]; *lptA* (100%, Q43H), responsible for LPS assembly [55]; and *hns* (66%, I70_L75 IS1 insertion), a global DNA-binding protein responsible for regulating metabolism and nutrient acquisition [56].

Several genes had significantly enriched mutations under both terrestrial and microgravity conditions. *trxA* is a processivity factor for T7 DNA polymerase and is a known essential gene for phage activity [32,57]. Deletions in *trxA* were significantly enriched in both conditions (terrestrial: 44.4%, K53_V56d deletion, microgravity: 100%, I76 frameshift), indicating the gene remains essential to the phage in microgravity. The same substitution in *ydcL* was significantly enriched in both conditions (I105K, terrestrial 100%, microgravity 33.9%). *ydcL* encodes a transcriptional regulator that triggers small, slow-growing persistor cell states, which could benefit bacteria during prolonged incubation conditions like those in this experiment [58,59].

Finally, intriguing indels were significantly enriched in *mlaA* in both conditions (Fig 4D). In each condition, two samples exhibited 6-bp deletions resulting in the loss of amino acids F44 and N45 (microgravity abundance: 95.5% and 16.5%; terrestrial abundance: 61.5% and 24.9%). In contrast, the third microgravity sample showed significant enrichment of a 6-bp insertion that added Asp and Phe, after N45 (30.2%, F46_N47ins), effectively inserting and repeating the same two amino acids deleted in the other samples.

mlaA encodes an outer membrane lipoprotein believed to remove mislocalized phospholipids from the outer membrane and shuttle them back to the inner membrane [60]. This gene has not yet been associated with changes in phage activity. A mutant with the same F44_N45 deletion has been characterized in *E. coli* MC4100 [61,62]. This mutation increases outer membrane permeability, phospholipid accumulation, and vesiculation—changes that could affect phage adsorption and potentially confer a competitive advantage. A prior study found that this deletion eventually led to bacterial cell death [62], but our results suggest this mutation may enhance bacterial survival under phage pressure. This discrepancy could also reflect differences in selection context, strain background, or the presence of suppressor mutations. Supporting this possibility, we also identified a significantly enriched frameshift-inducing deletion in *yhdP* (56.9%, L610 frameshift) in a microgravity sample that had the most abundant *mlaA* deletion. *yhdP* is involved in phospholipid transport to the outer membrane, and its loss has been shown to slow transport and reduce cell death in F44_N45 *mlaA* mutants [61], suggesting it may similarly enhance survivability in microgravity.

### Deep mutational scanning profiles beneficial substitutions in microgravity

Bacteria often resist phage predation by mutating or downregulating surface receptors essential for phage adsorption [26,63–65]. Microgravity-induced stress may amplify this response, altering the bacterial proteome, including phage receptor profiles [15,25,66]. Such changes can drive adaptive mutations in the phage RBP. To investigate these interactions, we examined how individual substitutions in the tip domain of the T7 RBP affect phage viability in microgravity.

The T7 RBP consists of six short non-contractile tails that form a homotrimer composed of a rigid shaft ending with a β-sandwich tip domain [67]. This domain is a key determinant of host recognition and interacts with host receptor LPS to position the phage for successful, irreversible binding [27–32,68]. We conducted comprehensive single-site saturation mutagenesis of the RBP tip domain, generating a library of 1,660 variants spanning residues 472–554 (based on PDB 4A0T). We then sequenced and compared mutational enrichment profiles following the 23-day selection under terrestrial and microgravity conditions.

**Fig 5:**
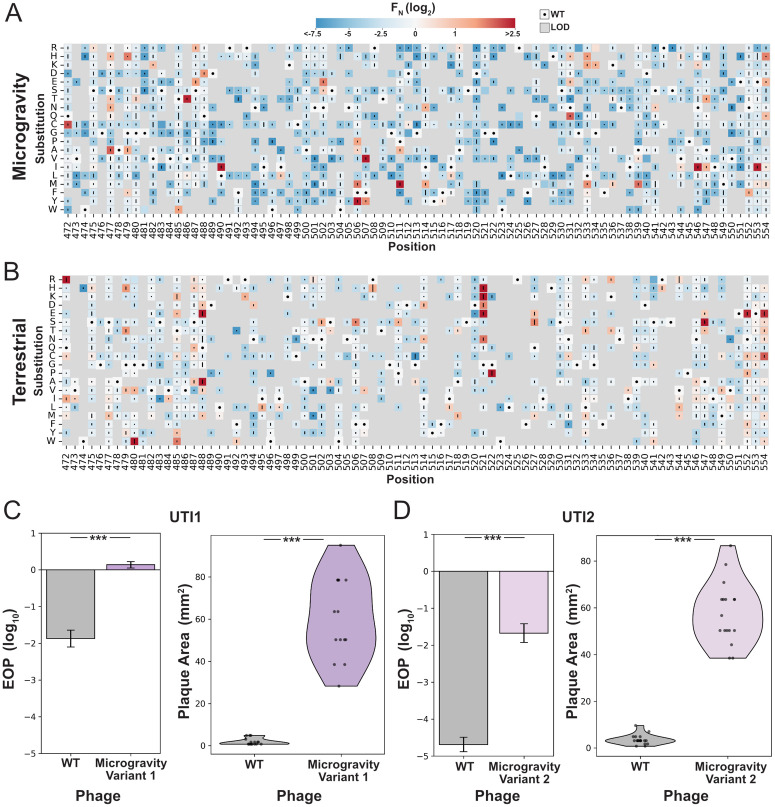
Deep mutational scanning reveals beneficial substitutions in microgravity. **(A, B)** Heatmaps of normalized functional scores (*F*_*N*_ log_2_) for all RBP substitutions after microgravity (A) and terrestrial (B) incubation. Scores shown on a blue to red gradient; Wildtype (WT, *F*_*N*_ log_2 _= 0) in white with a black dot; variants below the limit of detection (LOD) in gray. Line length represents standard deviation. Substitutions ordered top to bottom; residue positions (PDB 4A0T) shown left to right. **(C, D)** Efficiency of plating (EOP, left side) and violin plots of plaque area (right side) for (C) E. coli UTI1 and (D) E. coli UTI2 comparing wild type (WT, gray) and selected variant from the microgravity pool for that strain (right, purple). EOP Data shown as mean ± SD from three biological replicates, normalized to *E. coli* BL21. Significance vs. WT shown as *** (*p* < 0.001). The data underlying this Figure can be found in S1 Data.

We recovered phage DNA from each sample and scored each variant based on its relative abundance before and after selection (functional score, *F*) normalized to wildtype (normalized functional score, *F*_*N*_). Scores were averaged across replicates, and only variants present in at least two replicates were retained for analysis. Although significant dropout of low-performing variants was expected due to the extended incubation, we successfully determined scores for 51.2% (880) of variants in microgravity and 39% (648) in terrestrial conditions (Figs 5A, 5B, and S7A). Variant scores correlated well across replicates despite differences in phage titer and reflected multiple rounds of replication over the 23-day incubation period, suggesting that lower-titer samples underwent selection but subsequently lost viability (S7B–S7D Fig). On average phage variants were significantly more enriched after terrestrial incubation compared to microgravity (two-sample *t* test, Mann–Whitney *U*, *p* < 0.001) (S8A Fig). The wild-type phage was significantly depleted terrestrially compared to microgravity (terrestrial *F* = 0.58, microgravity *F* = 3.5, *p* < 0.01).

While variants that performed worse than wildtype (*F*_*N*_ < 0) tended to perform similarly between microgravity and terrestrial conditions (S8B Fig), enriched variants (*F*_*N*_ > 0) were highly divergent with no correlation between conditions (S8C Fig). Variants enriched in microgravity frequently contained methionine and isoleucine substitutions at interior positions facing the phage (Figs 5A and S8D), in contrast to our previous terrestrial results on this host [26]. Substitutions in these areas could influence the tip domain structure to facilitate adsorption with the host receptor in microgravity.

Under terrestrial conditions, top-scoring variants included positively charged substitutions facing the host, consistent with our previous findings on *E. coli* BL21 [26]. Additional enriched variants featured negatively charged substitutions (e.g., Q488E, G521D) and glycine substitutions (e.g., G480W, G522P) that may induce structural changes in the tip domain (Figs 5B and S8B). These variants were enriched only after prolonged incubation with *E. coli* BL21, suggesting that such substitutions may contribute to long-term infectivity on stationary-phase hosts—an effect not observed in shorter, nutrient-rich conditions.

Because variants enriched in microgravity were highly distinct from those identified under terrestrial conditions—both in this study and in our previous work—we next evaluated whether these substitutions could enhance phage activity terrestrially. If successful, these substitution patterns could be used to improve phage performance without exhaustively sampling the full combinatorial space of the gene. We constructed two combinatorial libraries, each comprising all possible combinations of 13 top-performing substitutions identified in microgravity (L490I, N502E, F506M, F506Y, F507V, F507Y, P511M, I514M, N531Q, L533K, L533M, A539M, N546I) or under terrestrial conditions (G521H, Q488A, Q488E, G521K, G522P, A547S, G521D, G521E, N502S, I495L, R542H, L533T, F506S). This strategy reduced a potential search space of over 10²¹ variants to fewer than 5,000 per library. Variants were synthesized in an oligo pool, assembled into an unbiased phage library using ORACLE, and passaged terrestrially on two clinically isolated *E. coli* strains (UTI1 and UTI2) that are resistant to wild-type T7 and are associated with urinary tract infections [69].

We evaluated these pools in efficiency of plating (EOP) experiments and compared their plaquing capability versus wildtype. The combinatorial pool from microgravity showed significant improvement in plaquing efficiency compared to wildtype and had substantially larger plaques, indicating the pool contained variants capable of significantly improving activity on these hosts (S9A and S9B Fig). The terrestrial library performed significantly worse or no better than wildtype. To confirm these results, we isolated individual plaques from the microgravity pool. From UTI1, we recovered a five-substitution variant (L490I, N502E, F507V, L533K, A539M; Variant 1), and from UTI2, a six-substitution variant (L490I, N502E, P511M, L533M, A539M, N546I; Variant 2). These variants demonstrated significantly higher EOP and produced significantly larger plaques on both UTI strains (Fig 5C and 5D). These findings support our hypothesis that microgravity-enriched substitutions can improve phage performance on terrestrial hosts. The extended incubation in microgravity revealed new mutational hotspots, enabling efficient navigation of sequence space to identify complex variant combinations with enhanced infectivity.

## Discussion

Phage–bacteria interactions play a critical role in shaping microbial ecosystems, but remain poorly understood in microgravity. In space, altered collision dynamics and bacterial physiological changes disrupt typical phage-host interplay. Characterizing these interactions provides insight into microbial adaptation in space and reveals novel genes and mechanisms with potential applications on Earth.

We found that phage replication in microgravity was significantly delayed -occurring sometime after the 4-hour time point -but ultimately successful by 23 days, indicating a markedly slower yet productive replication cycle. Phage stability also appeared more affected in microgravity than terrestrially, although this result would need to be confirmed with further experiments with more comprehensive timepoints. Numerous significantly enriched de novo mutations were identified in both phage and bacterial genes under microgravity and terrestrial conditions, suggesting strong selective pressures in both environments. In microgravity, structural genes *gp7.3*, *gp11*, and *gp12* emerged as particularly important, while enrichment of mutations in the non-structural gene *gp0.5* suggests its putative association with the host membrane may contribute more to phage fitness than previously recognized [43]. The overall distribution of mutations highlights genomic regions that warrant further investigation in future studies.

De novo mutations observed in the bacterial host were predominantly found in genes involved in outer membrane structure, stress response, and nutrient management. These findings are consistent with previous studies showing upregulation of similar gene classes in closely related *E. coli* strains under microgravity conditions. Such genes play key roles in managing environmental stress, regulating nutrient availability, and facilitating transmembrane transport in this unique setting [21,23,70–72]. The high frequency of mutations in these genes suggests they may reduce phage infectivity, offering bacteria an additional selective advantage. Bacteria had lower titer in 23-day samples in microgravity compared to terrestrial incubation, indicating mutations in these conditions may affect bacterial growth.

Results from the T7 RBP tip domain DMS library revealed significantly different selection patterns in microgravity compared to terrestrial incubation, both in this study and in our previous work [26]. Microgravity-enriched trends enabled efficient navigation of sequence space, leading to multi-substitution variants with significantly enhanced activity against uropathogenic *E. coli* under terrestrial conditions. Notably, combinatorial variants derived from terrestrial-enriched mutations failed to outperform wild-type, suggesting that the unique selective pressures of microgravity uncovered previously unrecognized functional regions with terrestrial relevance.

This study focused on a single non-motile strain of *E. coli*. Motile elements could potentially enhance fluid mixing, and future studies incorporating a broader range of bacterial strains would help clarify this effect. Additionally, the experimental design included several freeze–thaw cycles and a delay in processing, which reduced phage and bacterial viability. While some of these limitations are inherent to space-based research, minimizing freeze–thaw events and processing delays or assessing titers and sequencing directly on the ISS in future experiments could improve sample integrity and data quality.

Our study offers a preliminary look at how microgravity influences phage–host interactions. Exploring phage activity in non-terrestrial environments reveals novel genetic determinants of fitness and opens new avenues for engineering phages for terrestrial use. The success of this approach helps lays the groundwork for future phage research aboard the ISS.

## Methods

### Phage and bacterial strains

*T7 bacteriophage* was obtained from ATCC (ATCC BAA-1025-B2). The T7 DMS library used in this study was the same library stock generated in our previous work [26]. T7 acceptor phages used for ORACLE-based construction of the combinatorial libraries were also created as previously described [26]. *Escherichia coli BL21* was sourced from laboratory stocks. Uropathogenic *E. coli* strains UTI1 and UTI2 were provided by Dr. R. Welch (University of Wisconsin, Madison) and originate from a urinary tract infection isolate collection [69].

T7 phage was initially propagated on *E. coli* BL21 following receipt from ATCC and subsequently on appropriate hosts as described in specific experimental sections. All phage experiments were performed using Luria-Bertani (LB) media and the same culture conditions used for bacterial hosts. Phages were stored in LB at 4 °C for short-term use. For long-term storage, microbial samples were frozen at −80 °C in 100% LB media.

### Media and culture conditions

All bacterial strains were cultured in LB media consisting of 1% tryptone, 0.5% yeast extract, and 1% NaCl in deionized water. LB plates were supplemented with 1.5% agar, while top agar used for phage plating contained 0.5% agar. LB media was used for all experiments, including bacterial recovery and phage propagation. All incubations were carried out at 37 °C without shaking, in either terrestrial or microgravity environments as appropriate. These samples were incubated directly in cryovials and not transported to another container for incubation.

### Sample preparation and handling

Phage and bacterial stock titer were confirmed and samples were prepared by mixing 4 mL of *E. coli* BL21 in exponential phase (~1 × 10^8^ CFU/mL) with the appropriate amount of T7 phages in Rhodium Cryotubes. Samples were immediately frozen at −80 °C and shipped to NASA as described.

Asynchronous microgravity and terrestrial experiments are the norm for ISS experiments due to the uncertainty of scheduling. The initial planned time points for incubation were 1, 2, and 3 hours, and 25 days; however, actual time points were adjusted on the ISS to accommodate astronaut scheduling. Final incubation time points were 1, 2, and 4 hours, and 23 days. The duration of incubation aboard the ISS was recorded precisely, and terrestrial control samples were incubated for matching durations, based on the actual timepoints rather than the proposed schedule. This approach was necessary because real-time tracking of the samples was not possible, so microgravity and terrestrial samples could not be incubated in parallel accurately. Terrestrial samples are thus frozen for a longer duration than microgravity samples. After incubation samples were refrozen, shipped to our laboratory, and then thawed at 37 °C and immediately split for genomic DNA extraction, PCR for DMS, and titering of both phage and bacteria.

### Titering phage

For samples returned for processing, 1 mL of each sample was centrifuged at 16g for 1 min, and the supernatant was filtered through a 0.22 μm filter. To determine phage titer, titer was first estimated by spot plates and then confirmed by whole plate EOP assays. Samples were serially diluted (1:10 or 1:100) in LB to a final dilution of up to 10^−8^ in 1.5 mL microcentrifuge tubes. Spot assays were performed by mixing 250 μL of stationary-phase bacterial host with 3.5 mL of 0.5% top agar. The mixture was briefly vortexed and plated onto LB agar plates pre-warmed to 37 °C. Once the top agar solidified (~5 min), 1.5 μL of each phage dilution was spotted onto the plate in series. Plates were incubated at 37 °C and checked after 20–30 hours to estimate titer. Titers were then confirmed via full-plate plaque assays.

For whole-plate EOP assays, 400 μL of exponentially growing bacterial culture was mixed with 5–50 μL of diluted phage, aiming to achieve ~50 plaque-forming units (PFUs) per plate after overnight incubation. For phage susceptibility on the pre-incubation and 4-hour samples, bacteria was incubated after being directly sampled from the frozen stock for that sample. The phage–host mixture was briefly vortexed and centrifuged, then combined with 3.5 mL of 0.5% top agar. After a brief vortex, the mixture was immediately poured onto LB plates pre-warmed to 37 °C. Plates were allowed to solidify (~5 min), inverted, and incubated overnight. PFUs were counted after 20–30 hours, and final phage titers were calculated from these counts.

### Titering bacteria

Bacterial concentrations were determined via serial dilution (1:10 or 1:100 in LB) and plating. From each dilution, 100 μL was plated and spread using sterile beads to target ~50 colony-forming units (CFUs) per plate. Plates were incubated overnight at 37 °C and counted the following day. For *E. coli* BL21, three independent dilution series were performed to correlate OD_600_ values with CFU/mL and ensure accurate bacterial concentrations during phage mixing for experimental sample preparation.

### PCR and sequencing

All PCR reactions were performed using KAPA HiFi DNA Polymerase (Roche KK2101). The combinatorial library was generated using the ORACLE method, as previously described [26]. Cloning procedures followed manufacturer instructions unless otherwise specified.

For WGS, phage genomes were extracted using the Norgen Biotek Phage DNA Isolation Kit (Cat. 46800), and bacterial genomic DNA was extracted using the Norgen Biotek Bacterial Genomic DNA Isolation Kit (Cat. 17900). Genomic DNA libraries were prepared using the Illumina DNA Prep kit (Cat. 20060060) and sequenced on an Illumina NextSeq 1000 platform.

PCR reactions for amplification of the DMS and combinatorial libraries used 1 μL of undiluted phage lysate directly as template (DNA isolation is not required), with an extended denaturation step of 5 min at 95 °C. For low phage titers in DMS samples, PCR and next-generation sequencing failed using this approach, presumably because of reduced template in these samples. To overcome this, we concentrated all of the remaining volume of each sample (~2 mL) approximately 100-fold using Pierce Protein Concentrators PES, 10K MWCO (Cat. 88513) and used 3 μL of the concentrated sample per PCR reaction to enabling successful amplification and analysis. For plaque analysis on UTI strains, small plaque samples were picked directly and used as PCR template. Detailed cloning protocols are available upon request.

### General data analysis

Multiplicity of infection (MOI) was calculated by dividing the phage titer by the corresponding bacterial concentration. Initial MOI is calculated based on the bacteria and phage titer before being frozen for transit to the ISS. The MOI for the T7 DMS library was estimated using a helper plasmid, as described previously [26].

EOP values were calculated using *E. coli* BL21 as a reference host. EOP was defined as the phage titer on the test host divided by the titer on the reference host, followed by log₁₀ transformation. Values are reported as mean ± standard deviation (SD).

Deep sequencing was performed to evaluate phage populations as described previously [26]. Phage sequencing achieved an average depth of ~49,000× per base across the genome, enabling detection of low-abundance mutations. Bacterial sequencing depth averaged ~250× per base in phage-mixed samples and ~1,300× in phage-free samples, limiting mutation analysis in the former to more abundant variants.

WGS mutations were identified using Breseq [73]. For Fig 3, genes were grouped based on GO classifications [44,45]:

- Membrane-associated genes: GO:0016020 (Membrane), GO:0009103 (LPS biosynthesis), GO:0030288 (Outer membrane bound periplasmic space), GO:0042597 (Periplasmic space).
- Metabolism-associated genes: GO:0008152 (Metabolic process), GO:0019222 (Regulation of metabolic process).

### Statistical analysis

To evaluate whether non-synonymous de novo substitutions and frameshift mutations were significantly enriched compared to synonymous substitutions, we compared the frequency of each non-synonymous substitutions and frameshift (phage: >1% abundance; bacteria: >25% abundance) to the distribution of synonymous mutations using a one-sided Mann–Whitney *U* test with Benjamini–Hochberg false discovery rate (FDR) correction (scipy.stats.mannwhitneyu, statsmodels.stats.multitest.multipletests, method = ‘fdr_bh’,scipy V1.10.1, statsmodel v 0.14.0) [74]. Adjusted *p*-values < 0.05 were considered significant. This approach assumes that after 23 days of selection the distribution of synonymous substitutions approximates either a neutral baseline or reflects minimal selective pressure, with the benefit that if there is positive selection for synonymous substitutions there would be no increase in false positives using this method.

To determine whether non-synonymous de novo substitutions and frameshift mutations were more abundant in bacterial samples exposed to phage, we applied the Mann–Whitney *U* test (scipy.stats.mannwhitneyu, scipy V1.10.1) to compare mutation frequencies across groups [74,75]. Due to high detection limits in phage-mixed samples, we also performed left-censored data analysis using Kaplan–Meier survival curves (lifelines.KaplanMeierFitter, lifelines V0.27.8) and applied a log-rank test (lifelines.statistics.log-rank_test, lifelines V0.27.8) to assess significant differences in mutation distributions between groups [76–78].

To assess if the 4-hour bacterial population of mutations was significantly different from the pre-incubation condition, we performed Jenson–Shannon divergence (scipy.spatial.distance, jensenshannon, V1.10.1) and correlated results using Pearson R (scipy.stats, pearsonr, v1.10.1). To determine if the titer of wild-type T7 phage was significantly different between the pre-incubation and 4-hour bacterial population, we used a Welch’s *t* test (scipy.stats, ttest_ind_from_stats, v1.10.1).

Mutation density in phage genes was calculated by dividing the number of non-synonymous de novo substitutions and frameshift mutations by the length (in amino acids) of each protein product. To assess whether any gene had significantly higher mutation density, we compared individual gene densities to the condition-specific average using a one-tailed *t* test with Benjamini-Hochberg FDR correction (scipy.stats.ttest_1samp, statsmodels.stats.multitest.multipletests, method = ‘fdr_bh’, alternative = ‘greater’, scipy V1.10.1, statsmodel v 0.14.0). Additionally, one-tailed 95% confidence intervals were calculated using scipy.stats.t.ppf (scipy V1.10.1) and visualized in volcano plots in python [74].

### Structural visualization

Structural model images were generated using the PyMOL Molecular Graphics System, Version 3.0 (Schrödinger, LLC). *Gp7.3* structure was predicted using AlphaFold2 and ColabFold with MMseqs2, using the predicted structure with the highest confidence [79–81]. Electron Microscopy images were based on PDB 7BOX (gp11) and PDB 7BOY (gp12) [41]. A composite structure of the T7 portal–tail complex is shown from PDB 9JYZ [82]. Numbering for DMS and combinatorial library positions are based on PDB 4A0T [67].

## Supporting information

S1 Data Raw experimental data. (XLSX)

S1 Fig Mutation density confidence intervals and *t*-statistics. One-tailed 95% confidence intervals for mutation density (non-synonymous substitutions and frameshift count divided by protein length) for each phage gene after incubation in **(A)** microgravity and **(B)** after terrestrial incubation. Right panels show t-statistics and corresponding *p*-values from one-tailed t-tests with FDR correction. Genes with adjusted *p*-values <0.05 were considered significant. The data underlying this Figure can be found in S1 Data.
(TIFF)

S2 Fig Phage gene mutation abundance. Comparison of log_10_ abundance of de novo non-synonymous substitutions and frameshifts for phage genes not shown in Fig 2 after incubation in microgravity (left, dark green, Micro.) and terrestrially (right, light green, Terr.). No significant differences were detected, or data were too sparse to assess significance. Genes with mutations in only one condition show only that condition. The data underlying this Figure can be found in S1 Data.
(TIFF)

S3 Fig gp7.3 predicted structure. Predicted structure of *gp7.3* using Alphafold2. Positions with significantly enriched substitutions in microgravity are shown in blue. The unstructured region from G39 to Q55 related to in-frame deletions is shown in red.
(TIFF)

S4 Fig gp11 complex enriched mutations. **(A)** Electron microscopy structure of the *gp11* complex (PDB 7BOX) showing all 12 subunits. Mutations significantly enriched in microgravity are shown in blue; those enriched terrestrially in red. **(B)** Enlarged view of *gp11* with one subunit labeled, highlighting enriched mutations using the color scheme from (A). **(C)** Electron microscopy structure of the T7 portal-tail complex (PDB 9JYZ). *gp12* (portal protein) is shown in orange, *gp11* in green, *gp17* attachment in blue, and *gp8* (extending toward core proteins) in yellow. **(D)** Close-up of the interaction between R2 in *gp11* (green) and *R55* in gp17 (blue), with yellow dashed lines indicating contacts within 3.5 Å. R2 resides in an unstructured, flexible region that may interact with multiple *gp17* residues.
(TIFF)

S5 Fig gp12 complex enriched mutations. **(A)** Electron microscopy structure of the *gp12* complex (PDB 7BOY) showing all six subunits. Mutations significantly enriched in microgravity are shown in blue; those significantly enriched terrestrially in red. Positions are labeled on one representative subunit.
(TIFF)

S6 Fig Bacterial gene mutation survival curves, abundance, ranked allele frequency, and phage titer pre- and post-incubation. **(A, B)** Kaplan–Meier survival curves for bacterial non-synonymous substitutions and frameshift mutations after incubation (A) terrestrially or (B) in microgravity, with phage (light purple/light green) or without phage (dark purple/dark green). Shaded regions represent 95% confidence intervals. Survival probability reflects the proportion of mutations with abundance above the averaged limit of detection for each condition (log₁₀ −1 with phage, log₁₀ −2.5 without). P-values were calculated using log-rank tests. **(C)** Boxplots of log₁₀ abundance for non-synonymous substitutions and frameshift mutations after microgravity (left) or terrestrial (right) incubation, comparing conditions with (light shading) and without (dark shading) phage. Significance was assessed using a Mann–Whitney *U* test (*p < 0.05, **p < 0.01, ***p < 0.001), with “>” indicating the more abundant group. **(D)** Correlation of abundance mutations noted in preincubation condition (*X* axis) and from the 4-hour microgravity condition (4 hour, *Y* axis) with Pearson correlation shown. All mutations seen are distinct from de novo mutations described. **(E)** Ranked allele frequency spectra (Jenson–Shannon divergence = 0.0543 bits) showing frequency of mutations across each population in decreasing rank. **(F)** Phage titer of wild-type T7 grown on BL21 from pre-incubation bacterial sample and the 4-hour bacterial sample. There is no significant difference between these titers (*p* = 0.43). The data underlying this Figure can be found in S1 Data.
(TIFF)

S7 Fig Phage DMS replicate violin plots, titer and correlation. **(A)** Violin plots of DMS variant’s *F*_N_ (log_2_) scores across biological replicates (R1-3) in microgravity (left) and terrestrial (right) conditions. **(B)** Phage titer (log_10_ PFU) for DMS replicates 1, 2, and 3 (R1, R2, and R3) after microgravity (left, purple) and terrestrial (right, green) incubation, shown as mean ± SD. **(C, D)** Correlation plot of DMS variants F_N_ (log_2_) score between replicates (R1-3) in (C) microgravity and (D) terrestrial conditions. Pearson’s r is displayed on the bottom right of each plot. The data underlying this Figure can be found in S1 Data.
(TIFF)

S8 Fig Terrestrial and microgravity phage DMS comparison and gp17 structure with enriched mutations. **(A)** Violin plots of average *F*_*N*_ (log_2_) for DMS variants in microgravity (left, purple) and terrestrial (right, green) conditions. Significance indicated as *** (*p* < 0.001). **(B)** Correlation plot of *F*_*N*_ (log₂) scores for all DMS variants between microgravity and terrestrial conditions. Pearson’s *r* displayed in the bottom right. **(C)** Correlation of enriched (*F*_*N*_ (log_2_) > 0) variants between conditions. Pearson’s *r* shown bottom left. **(D)** Crystal structure and secondary structure topology of the RBP tip domain (PDB: 4A0T), with substitutions enriched in microgravity (purple) or terrestrial (green) conditions highlighted. Two views are shown for clarity with a 145° rotation. The data underlying this Figure can be found in S1 Data.
(TIFF)

S9 Fig Efficiency of plating (EOP) results comparing wild type, terrestrial, and microgravity combinatorial pools on *Escherichia coli* UTI strains. **(A, B)** EOP results on (A) *E. coli* UTI1 and (B) *E. coli* UTI2 comparing wild type (WT, left, gray), terrestrial combinatorial pool (middle, green), and microgravity combinatorial pool (right, purple). Data shown as mean ± SD from three biological replicates, normalized to *E. coli* BL21. Significance versus WT shown as * (*p* < 0.05), *** (*p* < 0.001), or n.s. (not significant). The data underlying this Figure can be found in S1 Data.
(TIFF)

## Abbreviations

CFUs: colony-forming units
DMS: deep mutational scanning
FDR: false discovery rate
GO: Gene Ontology
ISS: International Space Station
LB: Luria-Bertani
MOI: multiplicity of infection
PFUs: plaque-forming units
RBP: receptor binding protein
SD: standard deviation
WGS: whole-genome sequencing
