# Supplementary figures and images for: Microgravity reshapes bacteriophage–host coevolution aboard the International Space Station
Source: PLoS Biol. 2026 Jan 13;24(1):e3003568. doi: 10.1371/journal.pbio.3003568 (PMC12798971; doi:10.1371/journal.pbio.3003568)

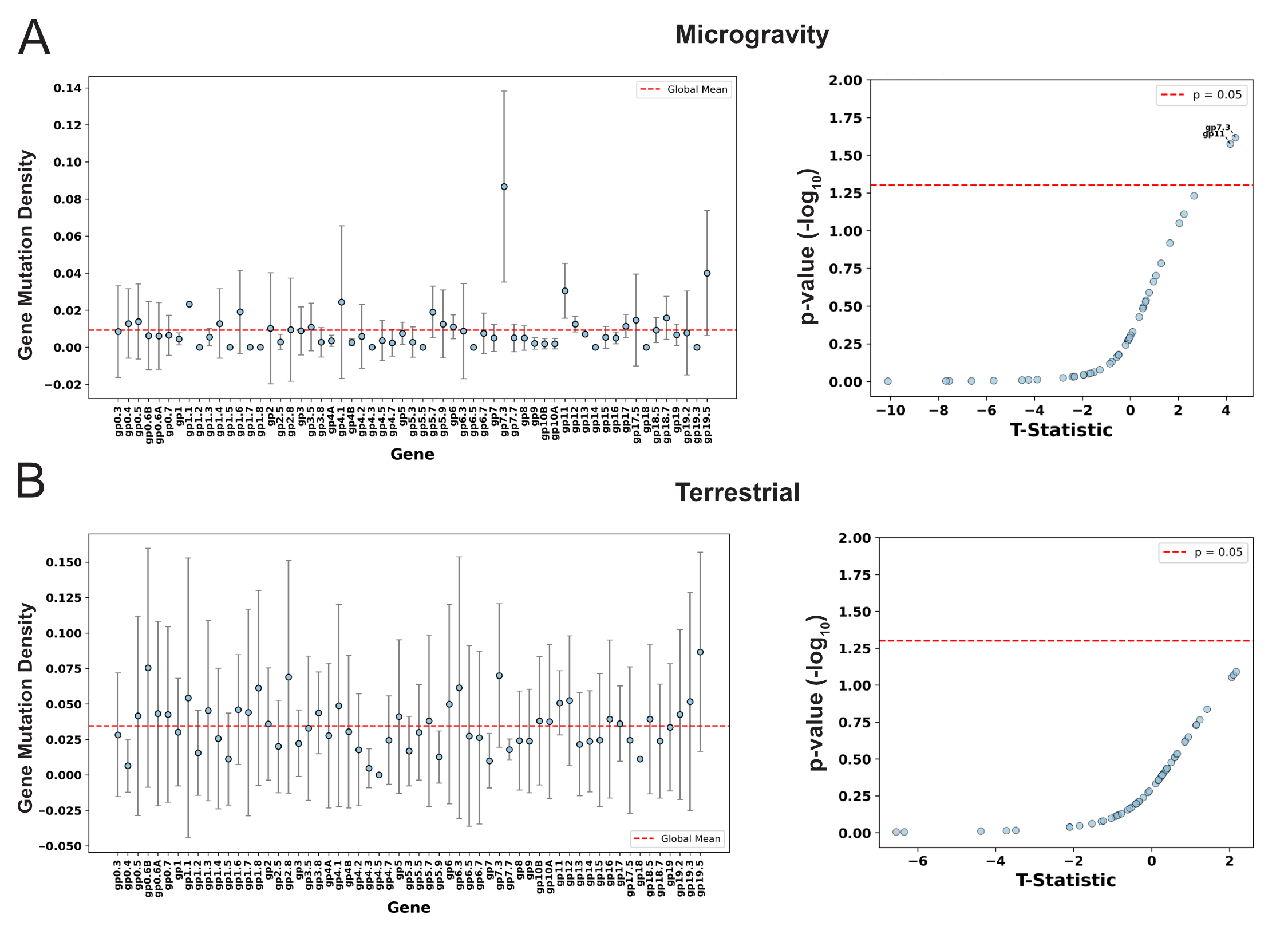

Supplement: S1 Fig — One-tailed 95% confidence intervals for mutation density (non-synonymous substitutions and frameshift count divided by protein length) for each phage gene after incubation in (A) microgravity and (B) after terrestrial incubation. Right panels show t-statistics and corresponding p-values from one-tailed t-tests with FDR correction. Genes with adjusted p-values <0.05 were considered significant. The data underlying this Figure can be found in S1 Data. (TIFF) [file pbio.3003568.s002.tiff]

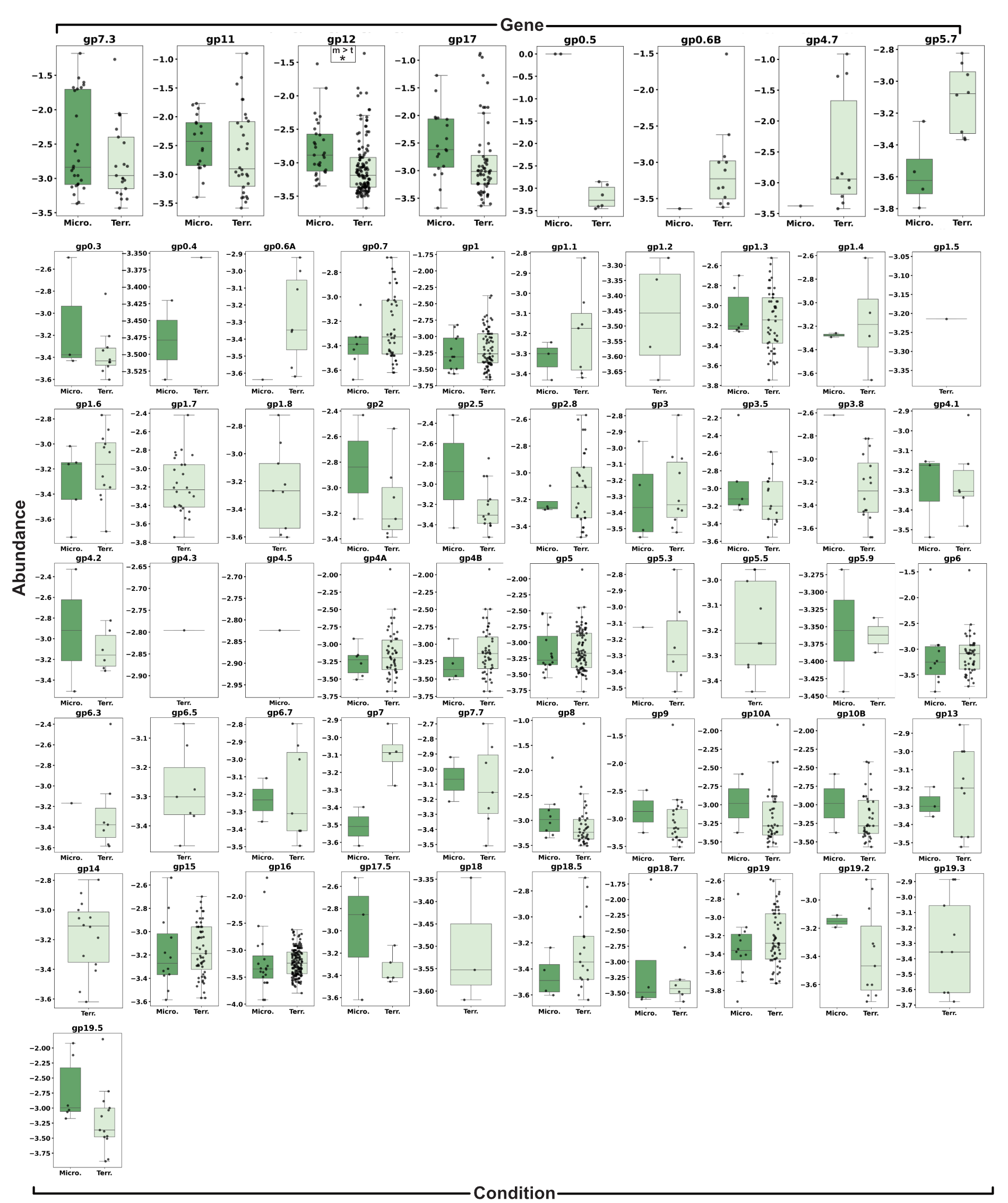

Supplement: S2 Fig — Comparison of log10 abundance of de novo non-synonymous substitutions and frameshifts for phage genes not shown in Fig 2 after incubation in microgravity (left, dark green, Micro.) and terrestrially (right, light green, Terr.). No significant differences were detected, or data were too sparse to assess significance. Genes with mutations in only one condition show only that condition. The data underlying this Figure can be found in S1 Data. (TIFF) [file pbio.3003568.s003.tiff]

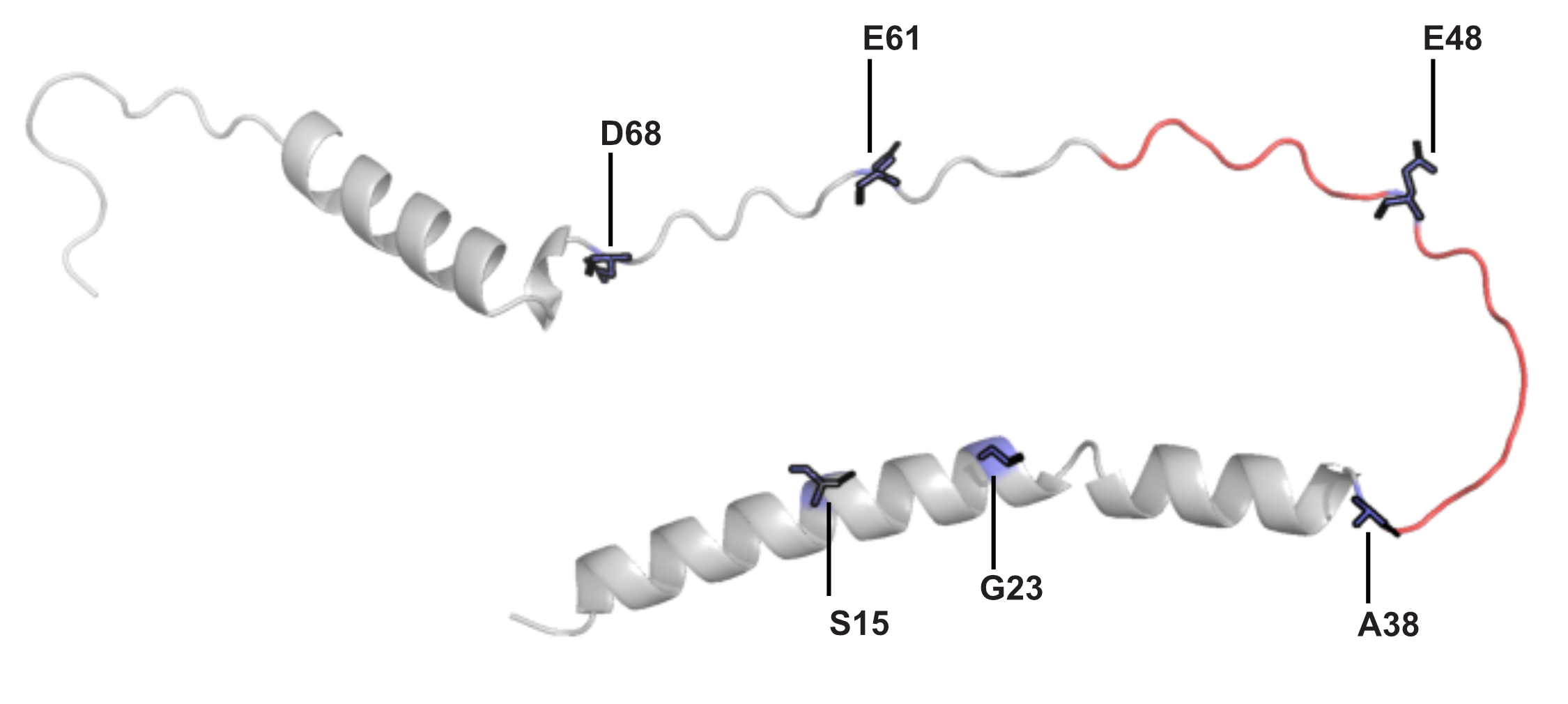

Supplement: S3 Fig — Predicted structure of gp7.3 using Alphafold2. Positions with significantly enriched substitutions in microgravity are shown in blue. The unstructured region from G39 to Q55 related to in-frame deletions is shown in red. (TIFF) [file pbio.3003568.s004.tiff]

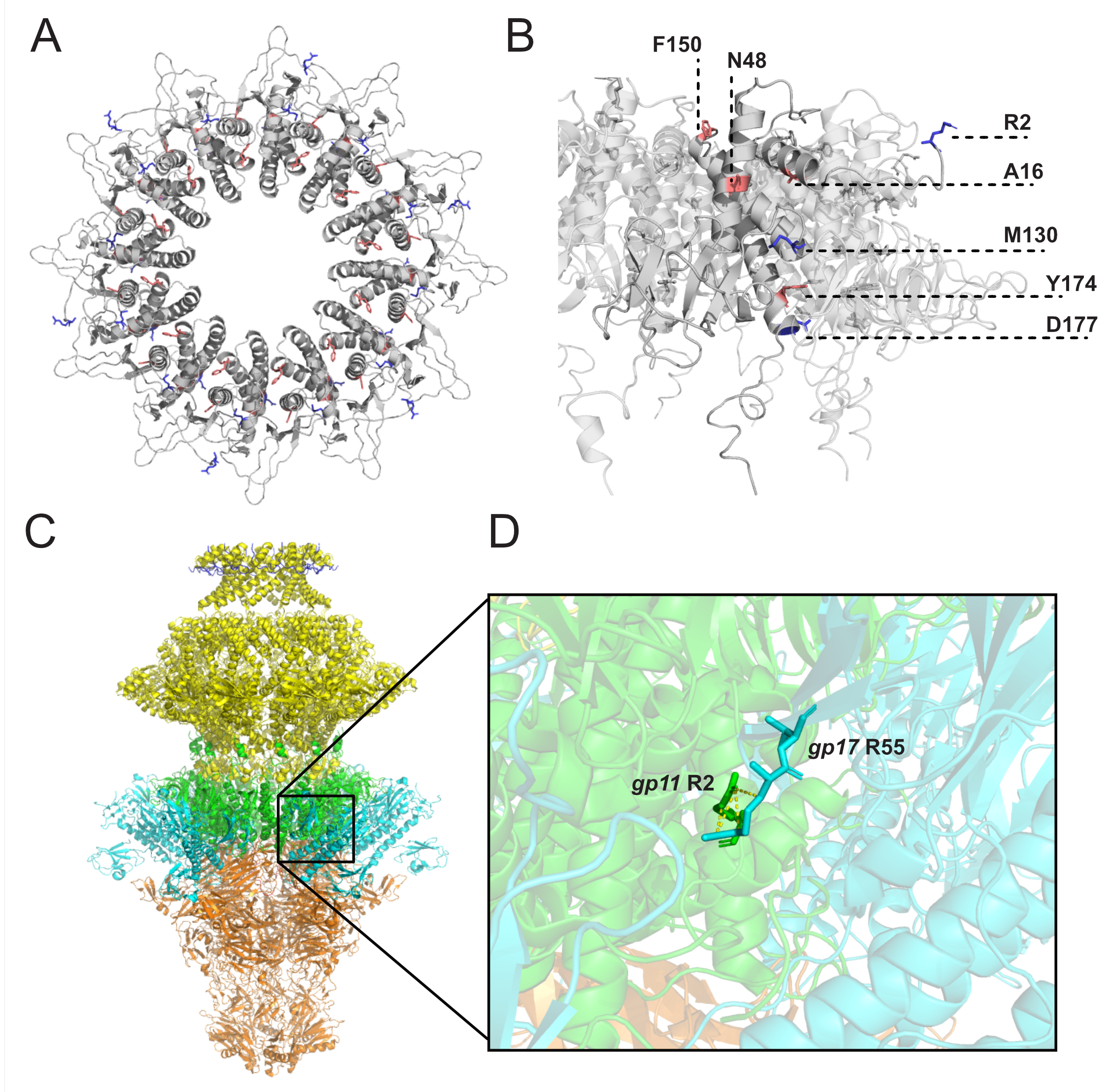

Supplement: S4 Fig — (A) Electron microscopy structure of the gp11 complex (PDB 7BOX) showing all 12 subunits. Mutations significantly enriched in microgravity are shown in blue; those enriched terrestrially in red. (B) Enlarged view of gp11 with one subunit labeled, highlighting enriched mutations using the color scheme from (A). (C) Electron microscopy structure of the T7 portal-tail complex (PDB 9JYZ). gp12 (portal protein) is shown in orange, gp11 in green, gp17 attachment in blue, and gp8 (extending toward core proteins) in yellow. (D) Close-up of the interaction between R2 in gp11 (green) and R55 in gp17 (blue), with yellow dashed lines indicating contacts within 3.5 Å. R2 resides in an unstructured, flexible region that may interact with multiple gp17 residues. (TIFF) [file pbio.3003568.s005.tiff]

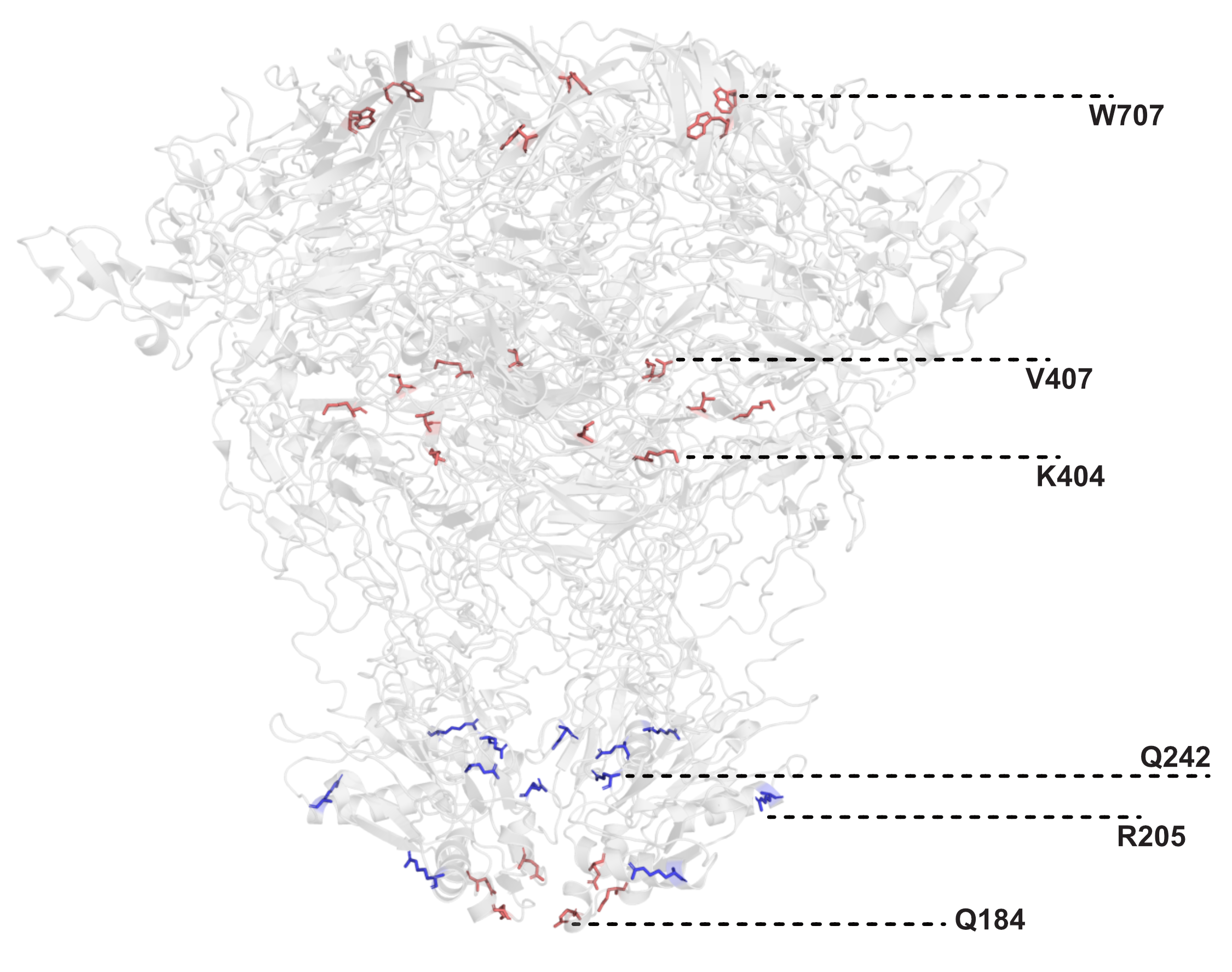

Supplement: S5 Fig — (A) Electron microscopy structure of the gp12 complex (PDB 7BOY) showing all six subunits. Mutations significantly enriched in microgravity are shown in blue; those significantly enriched terrestrially in red. Positions are labeled on one representative subunit. (TIFF) [file pbio.3003568.s006.tiff]

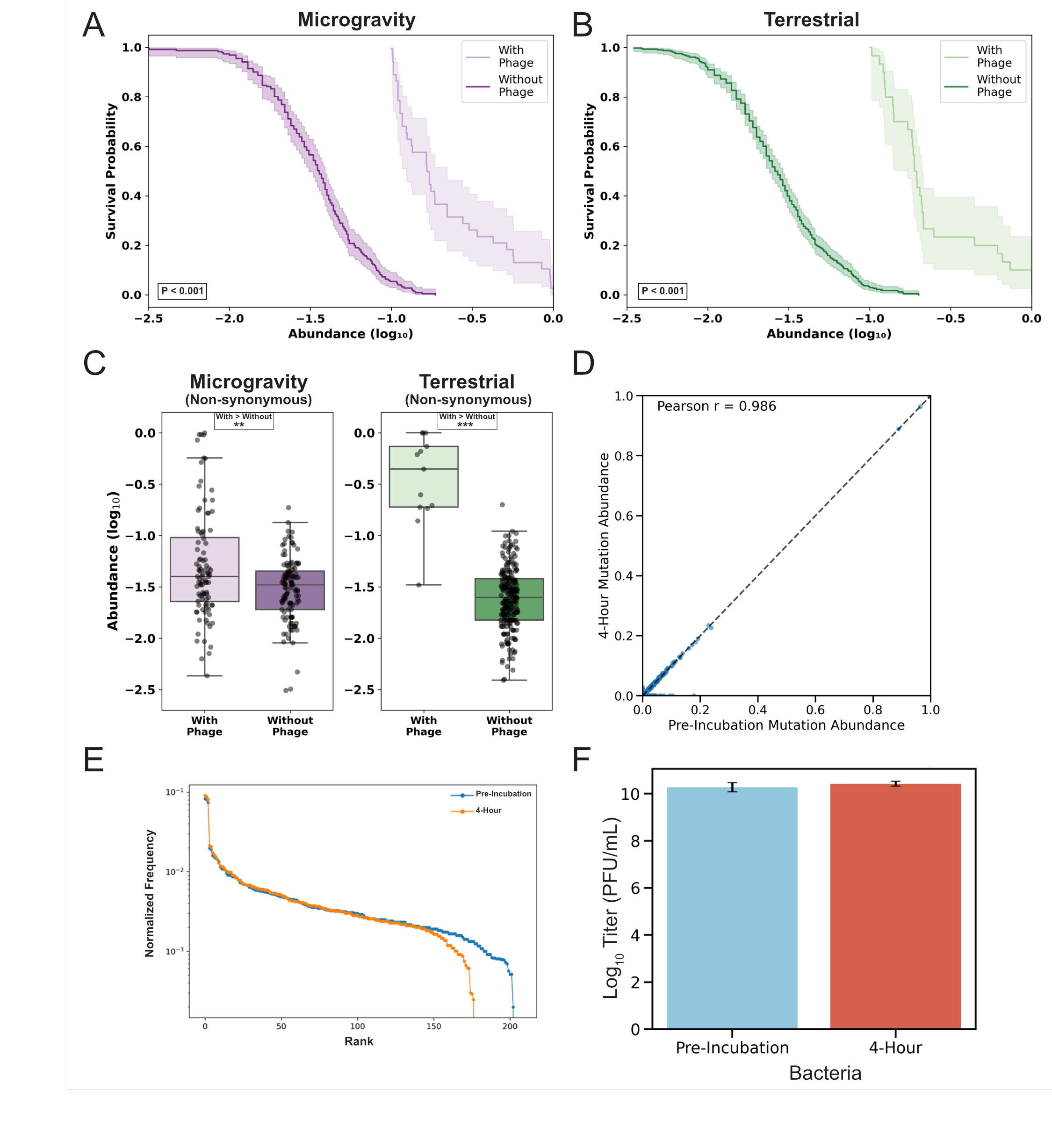

Supplement: S6 Fig — (A, B) Kaplan–Meier survival curves for bacterial non-synonymous substitutions and frameshift mutations after incubation (A) terrestrially or (B) in microgravity, with phage (light purple/light green) or without phage (dark purple/dark green). Shaded regions represent 95% confidence intervals. Survival probability reflects the proportion of mutations with abundance above the averaged limit of detection for each condition (log₁₀ −1 with phage, log₁₀ −2.5 without). P-values were calculated using log-rank tests. (C) Boxplots of log₁₀ abundance for non-synonymous substitutions and frameshift mutations after microgravity (left) or terrestrial (right) incubation, comparing conditions with (light shading) and without (dark shading) phage. Significance was assessed using a Mann–Whitney U test (*p < 0.05, **p < 0.01, ***p < 0.001), with “>” indicating the more abundant group. (D) Correlation of abundance mutations noted in preincubation condition (X axis) and from the 4-hour microgravity condition (4 hour, Y axis) with Pearson correlation shown. All mutations seen are distinct from de novo mutations described. (E) Ranked allele frequency spectra (Jenson–Shannon divergence = 0.0543 bits) showing frequency of mutations across each population in decreasing rank. (F) Phage titer of wild-type T7 grown on BL21 from pre-incubation bacterial sample and the 4-hour bacterial sample. There is no significant difference between these titers (p = 0.43). The data underlying this Figure can be found in S1 Data. (TIFF) [file pbio.3003568.s007.tiff]

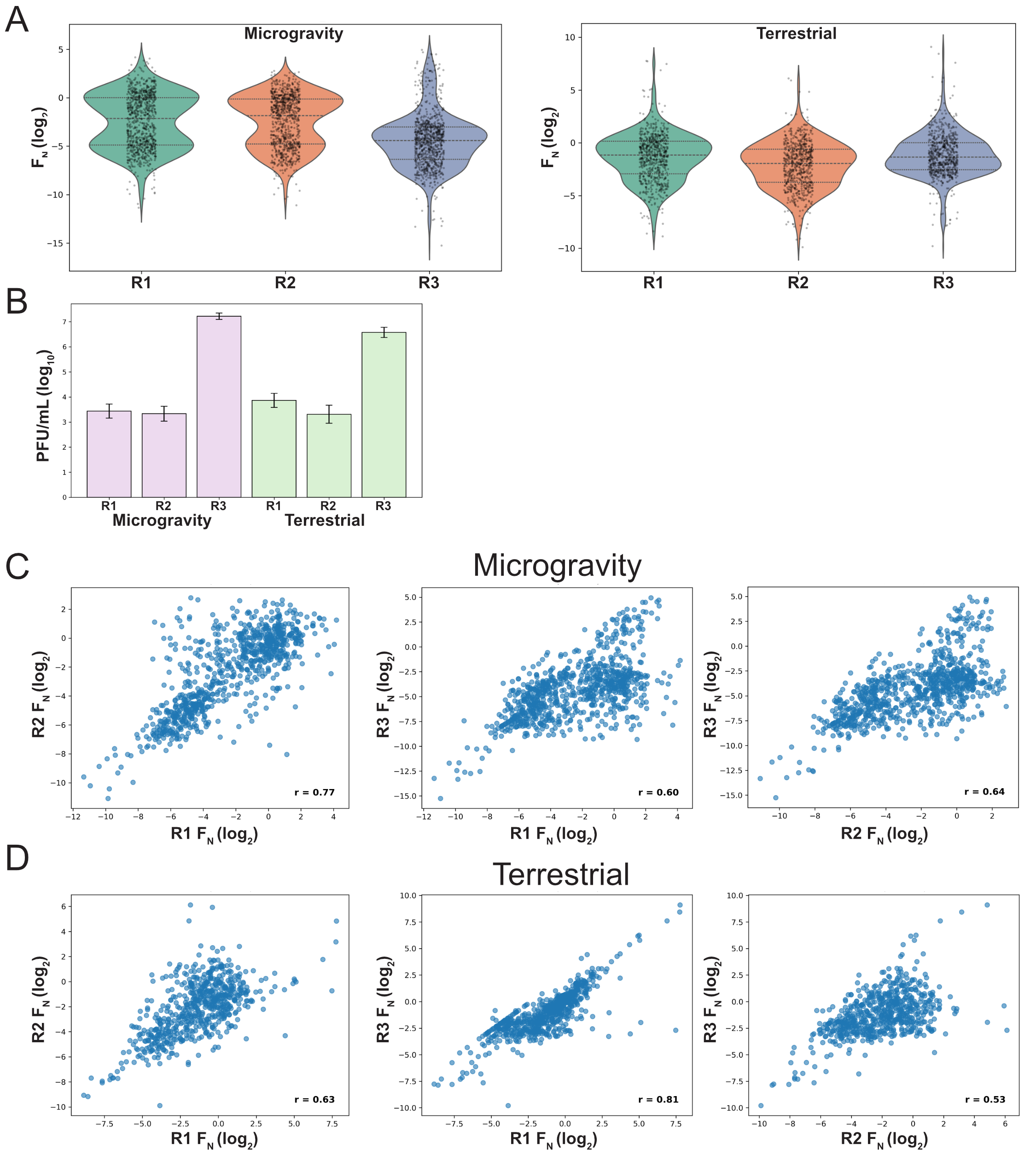

Supplement: S7 Fig — (A) Violin plots of DMS variant’s FN (log2) scores across biological replicates (R1-3) in microgravity (left) and terrestrial (right) conditions. (B) Phage titer (log10 PFU) for DMS replicates 1, 2, and 3 (R1, R2, and R3) after microgravity (left, purple) and terrestrial (right, green) incubation, shown as mean ± SD. (C, D) Correlation plot of DMS variants FN (log2) score between replicates (R1-3) in (C) microgravity and (D) terrestrial conditions. Pearson’s r is displayed on the bottom right of each plot. The data underlying this Figure can be found in S1 Data. (TIFF) [file pbio.3003568.s008.tiff]

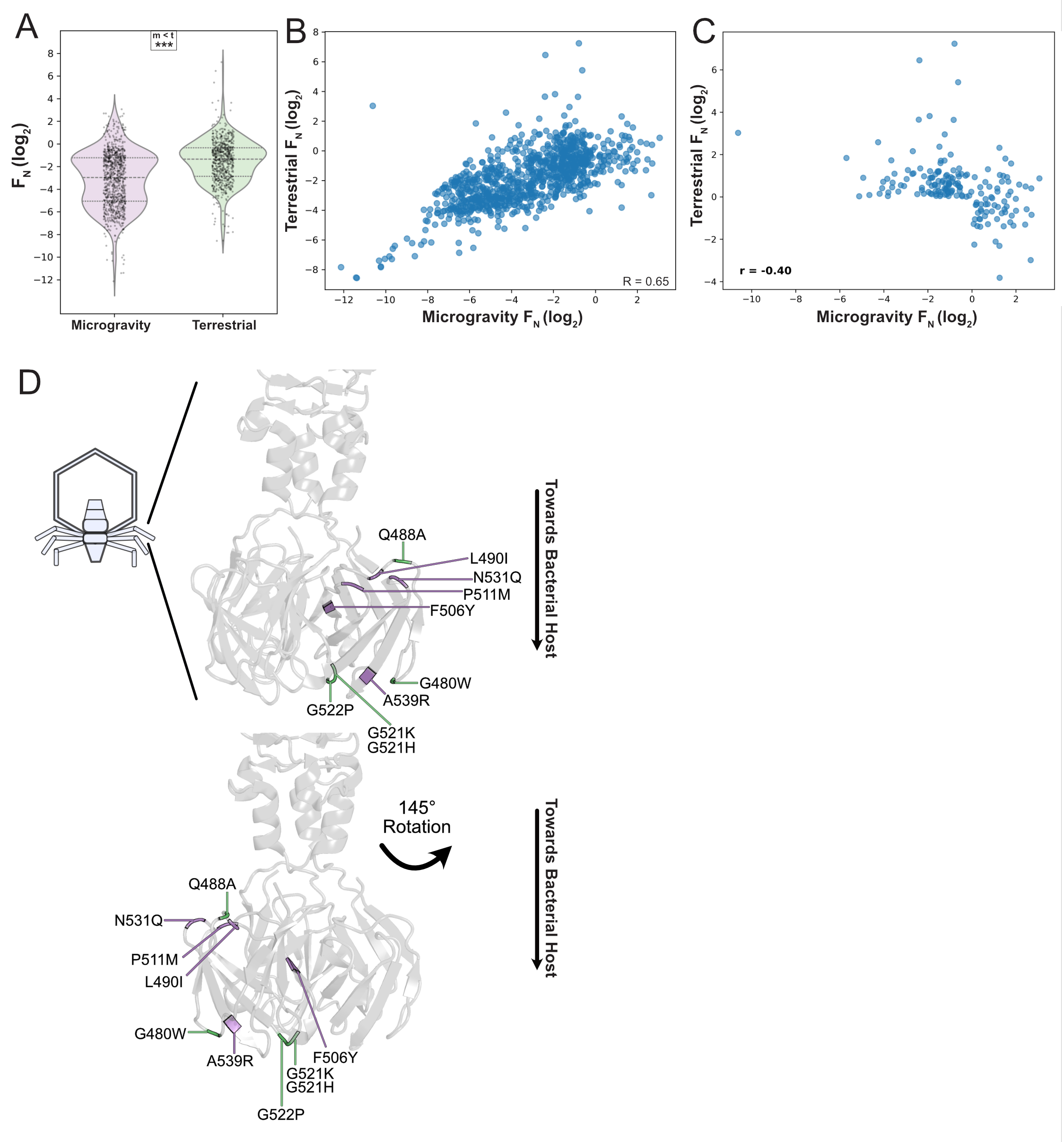

Supplement: S8 Fig — (A) Violin plots of average FN (log2) for DMS variants in microgravity (left, purple) and terrestrial (right, green) conditions. Significance indicated as *** (p < 0.001). (B) Correlation plot of FN (log₂) scores for all DMS variants between microgravity and terrestrial conditions. Pearson’s r displayed in the bottom right. (C) Correlation of enriched (FN (log2) > 0) variants between conditions. Pearson’s r shown bottom left. (D) Crystal structure and secondary structure topology of the RBP tip domain (PDB: 4A0T), with substitutions enriched in microgravity (purple) or terrestrial (green) conditions highlighted. Two views are shown for clarity with a 145° rotation. The data underlying this Figure can be found in S1 Data. (TIFF) [file pbio.3003568.s009.tiff]

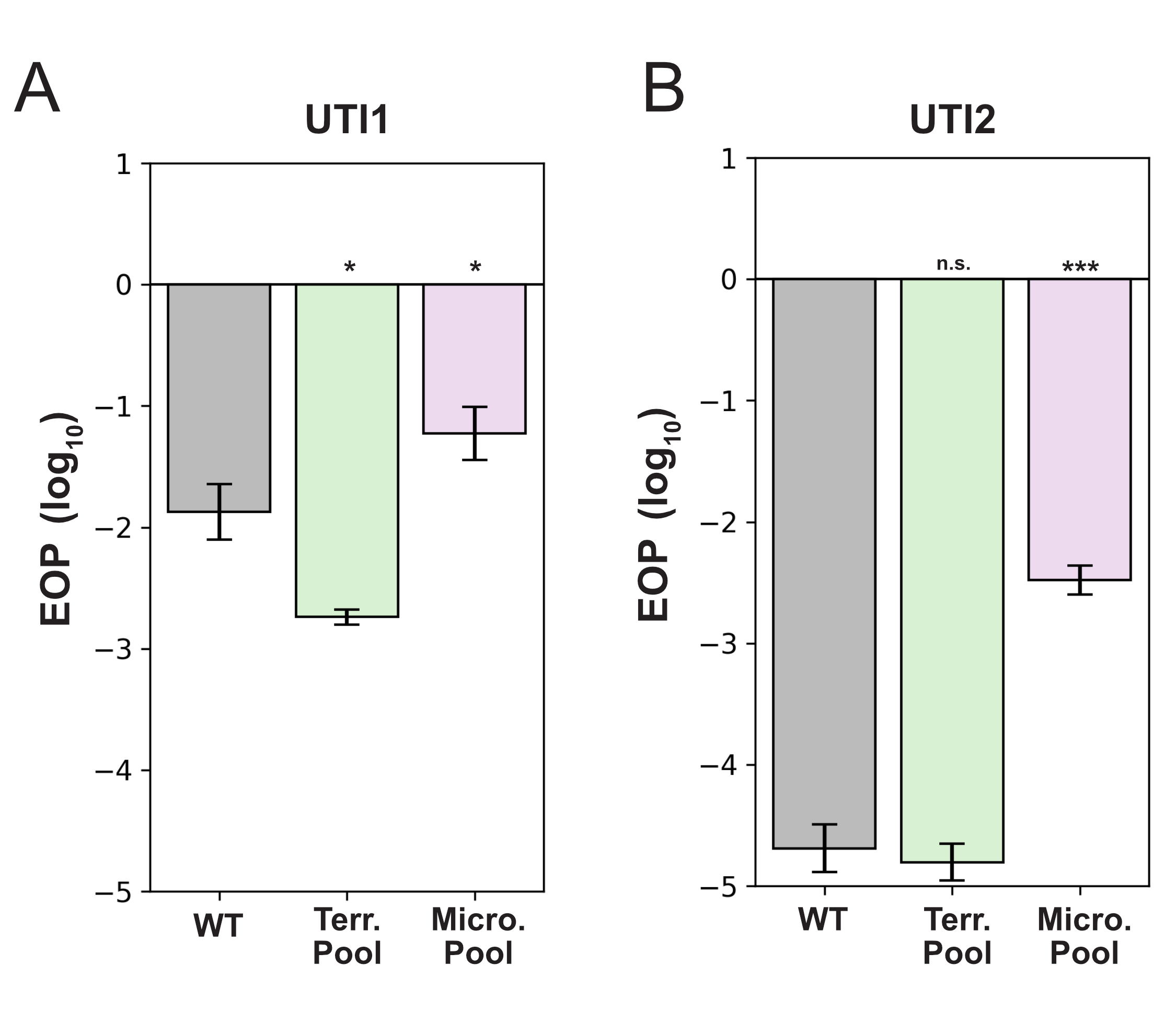

Supplement: S9 Fig — (A, B) EOP results on (A) E. coli UTI1 and (B) E. coli UTI2 comparing wild type (WT, left, gray), terrestrial combinatorial pool (middle, green), and microgravity combinatorial pool (right, purple). Data shown as mean ± SD from three biological replicates, normalized to E. coli BL21. Significance versus WT shown as * (p < 0.05), *** (p < 0.001), or n.s. (not significant). The data underlying this Figure can be found in S1 Data. (TIFF) [file pbio.3003568.s010.tiff]
